# Cytotoxicity and Multi-Enzyme Inhibition of *Nepenthes miranda* Stem Extract on H838 Human Non-Small Cell Lung Cancer Cells and RPA32, Elastase, Tyrosinase, and Hyaluronidase Proteins

**DOI:** 10.3390/plants13060797

**Published:** 2024-03-11

**Authors:** Ching-Yi Lee, Yu-Cheng Chen, Yen-Hua Huang, Yi Lien, Cheng-Yang Huang

**Affiliations:** 1 Department of Internal Medicine, Tao Yuan General Hospital, Ministry of Health and Welfare, Taoyuan 330, Taiwan; 2 Department of Biomedical Sciences, Chung Shan Medical University, Taichung City 402, Taiwan; 3 Department of Biological Sciences, Purdue University, West Lafayette, IN 47907, USA; 4 Department of Medical Research, Chung Shan Medical University Hospital, Taichung City 402, Taiwan

**Keywords:** *Nepenthes*, anticancer, H838 lung carcinoma, anti-skin aging, elastase, RPA, GC–MS analysis, AntoDock, stigmast-5-en-3-ol, plumbagin

## Abstract

The carnivorous pitcher plants of the genus *Nepenthes* have long been known for their ethnobotanical applications. In this study, we prepared various extracts from the pitcher, stem, and leaf of *Nepenthes miranda* using 100% ethanol and assessed their inhibitory effects on key enzymes related to skin aging, including elastase, tyrosinase, and hyaluronidase. The cytotoxicity of the stem extract of *N. miranda* on H838 human lung carcinoma cells were also characterized by effects on cell survival, migration, proliferation, apoptosis induction, and DNA damage. The cytotoxic efficacy of the extract was enhanced when combined with the chemotherapeutic agent 5-fluorouracil (5-FU), indicating a synergistic effect. Flow cytometry analysis suggested that the stem extract might suppress H838 cell proliferation by inducing G2 cell cycle arrest, thereby inhibiting carcinoma cell proliferation. Gas chromatography–mass spectrometry (GC–MS) enabled the tentative identification of the 15 most abundant compounds in the stem extract of *N. miranda*. Notably, the extract showed a potent inhibition of the human RPA32 protein (huRPA32), critical for DNA replication, suggesting a novel mechanism for its anticancer action. Molecular docking studies further substantiated the interaction between the extract and huRPA32, highlighting bioactive compounds, especially the two most abundant constituents, stigmast-5-en-3-ol and plumbagin, as potential inhibitors of huRPA32’s DNA-binding activity, offering promising avenues for cancer therapy. Overall, our findings position the stem extract of *N. miranda* as a promising source of natural compounds for anticancer therapeutics and anti-skin-aging treatments, warranting further investigation into its molecular mechanisms and potential clinical applications.

## 1. Introduction

*Nepenthes*, a plant traditionally utilized in folk medicine, has been employed to remedy various ailments, including stomachaches and fevers [1]. Owing to its longstanding use in ethnomedicine, extracts from *Nepenthes* are considered relatively safe for pharmaceutical applications, potentially offering minimal side effects for human consumption. Consequently, the exploration and development of additional therapeutic uses are warranted.

Cancer, characterized by uncontrolled cell growth and the potential for tumors to spread locally or systemically, remains one of the world’s deadliest diseases [2,3,4]. WHO reports estimate around 20 million new cases and 10 million fatalities from cancer in 2023. Notably, lung cancer ranks among the primary causes of these fatalities. Non-small cell lung cancer (NSCLC), constituting approximately 85% of lung cancer cases, is commonly treated through surgery, chemotherapy, radiation, and targeted therapy. However, NSCLCs often show limited responsiveness to chemotherapy [5,6]. Recently, natural compounds are increasingly explored as potential anti-lung cancer agents and alternative treatment options [7,8,9,10]. These phytochemicals may also offer solutions to challenges like drug resistance, a common issue in lung cancer treatment [11]. Accordingly, this study assesses the cytotoxic effects of *Nepenthes miranda* extract on the viability, migration, proliferation, DNA damage, and apoptosis of human NSCLC H838 carcinoma cells. A notable advantage of using natural extracts against cancer cells lies in their multi-targeted modes of action [12]. Furthermore, we also investigate the combined effects of *N. miranda* extract with the clinical anticancer drug 5-fluorouracil (5-FU) to determine the presence of any synergistic effects in combating NSCLC.

The skin, the most extensive and intricate organ in the human body, serves as a crucial barrier protecting against external elements [13]. Its appearance significantly influences social interactions, with youthful and healthy skin often perceived positively in social contexts [14,15]. As we age, our skin undergoes changes that can profoundly affect our overall health and quality of life, manifesting as thinness, dryness, reduced elasticity, rough texture, wrinkles, and dark spots [16,17]. Accordingly, the continual discovery of anti-aging agents holds substantial value. Plant-based compounds, particularly secondary metabolites and whole plant extracts, have been extensively studied for their anti-aging properties [18]. Plants are rich in compounds like polyphenols, renowned for their potent antioxidant properties, combating aging and photodamage. Isolated polyphenols from green tea, such as catechin and epigallocatechin gallate (EGCG), are known to target aging-associated enzymes like elastase [19]. Other natural compounds, including quercetin (Que) and myricetin (Myr), have been identified as inhibitors of tyrosinase [20] and hyaluronidase [21]. *Nepenthes* thrives in extended exposure to bright sunlight, essential for its optimal growth. Given the association between UV light exposure and photodamage, skin aging, and skin cancer, exploring the anti-skin aging properties of *Nepenthes* extract is crucial. Accordingly, this study also aims to investigate this potential by examining the extract’s inhibitory effects on enzymes associated with skin aging, specifically elastase, tyrosinase, and hyaluronidase. Extracts from the *Nepenthes* plant, such as those from *N. miranda* [22,23], are noted for their high polyphenol content and significant antioxidant activity, suggesting their potential in anti-aging applications. Therefore, it is worth determining whether *N. miranda* extracts can specifically inhibit enzymes associated with the aging process.

Previously, the single-stranded DNA (ssDNA)-binding protein (SSB) [24], a pivotal player in DNA metabolism processes such as replication, repair, recombination, and replication fork restart in bacterial cells [25], was pinpointed as a molecular target inhibited by the *N. miranda* extract [26]. SSB, indispensable for bacterial DNA replication and cell reproduction, functions as a homotetramer where four oligonucleotide/oligosaccharide-binding (OB) folds create a DNA-binding domain [27,28,29]. The inhibition of SSB holds pharmacological promise for targeting bacterial pathogens [30,31,32]. Replication protein A (RPA) is a eukaryotic counterpart to bacterial SSB and exhibit similar mechanistic roles [33]. RPA, also an OB-fold protein, consists of a heterotrimer comprised of RPA70, RPA32, and RPA14 subunits, each capable of independent function. RPA32, in particular, binds ssDNA and interacts with various genome maintenance proteins, including RAD52, TIPIN, XPA, UNG2, and SMARCAL1 [34]. RPA32 shares structural and functional traits with alpha-accessory factor-44 (AAF-44), regulating DNA replication and enhancing DNA primase and polymerase-alpha activities [35]. Inhibition of RPA triggers replication stress, represses tumor growth [36], and is linked to cell death post-irradiation [37]. In addition, the decrease of RPA32 foci formation plays a role in suppressing NSCLC [38]. Considering that normal human cells do not require continuous DNA synthesis, an inhibitor targeting RPA32 could demonstrate selective cytotoxicity towards rapidly proliferating cells in need of genome replication. Similar to the case of inhibiting pathogenic SSB [26], *N. miranda* extract was investigated as a potential inhibitor of RPA32.

The *Nepenthes* plant, traditionally used in folk medicine, may offer novel therapeutic applications. This study broadens its medicinal scope to include cytotoxicity against H838 human NSCLC carcinoma cells, anti-skin aging, and RPA32 inhibition, employing a stem extract of *N. miranda* extracted with 100% ethanol. Ethanol, being a safer solvent for human use, is a preferable option for extractions. Using gas chromatography–mass spectrometry (GC–MS) and the docking program AutoDock Vina, we tentatively identified the chemical constituents of the extract and predicted the binding interactions of these compounds with RPA32. The results of this study collectively suggest that *N. miranda* holds potential pharmacological benefits, warranting further exploration for therapeutic applications.

## 2. Results

### 2.1. Anti-Elastase Activity of N. miranda Extract

**Figure 1:**
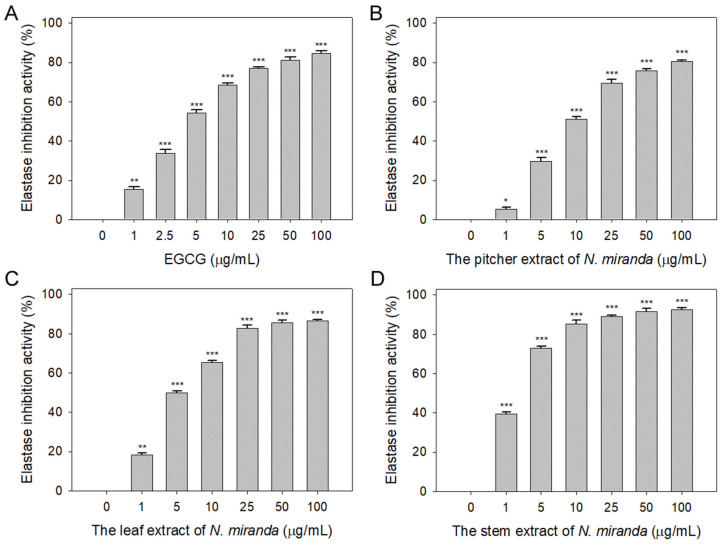
Inhibition of elastase activity by *N. miranda* extract. The inhibitory effects were demonstrated on elastase activity by (**A**) EGCG and *N. miranda* extracts from the (**B**) pitcher, (**C**) leaf, and (**D**) stem. AAAPVN was utilized as the substrate. EGCG was employed as a positive control, while 10% DMSO was used as a negative control (indicating 0 μg/mL of *N. miranda* extract). Levels of statistical significance are denoted by * *p* < 0.05, ** *p* < 0.01, and *** *p* < 0.001 when compared to the control group.

The role of elastin, which constitutes 2–4% of the extracellular matrix, is crucial in maintaining skin elasticity and hydration [39]. Elastase, a protease, is responsible for breaking protein bonds and degrading them into polypeptides or amino acids. Dysregulation in the extracellular release of elastase can lead to the degradation of elastin and other matrix components, causing damage to epithelial cells. The potential of *Nepenthes* plant extracts in inhibiting elastase activity has not been previously explored and warrants investigation. In this study, we assessed the elastase inhibitory activity of extracts from *N. miranda*’s pitcher, leaf, and stem (0–100 μg/mL) obtained using 100% ethanol. The assay employed N-Succinyl-Ala-Ala-Ala-*p*-nitroanilide (AAAPVN) as the substrate, with epigallocatechin gallate (EGCG) serving as a positive control (0–100 μg/mL). At a concentration of 5 μg/mL, EGCG (Figure 1A), and the extracts from the pitcher (Figure 1B), leaf (Figure 1C), and stem (Figure 1D) of *N. miranda* displayed inhibition levels of 54%, 29%, 50%, and 73%, respectively. The half-maximal inhibitory concentrations (IC_50_) were determined to be 4.41 ± 0.34 μg/mL for EGCG, 8.66 ± 1.15 μg/mL for the pitcher, 5.31 ± 0.31 μg/mL for the leaf, and 2.29 ± 0.68 μg/mL for the stem extract (Table 1). These findings reveal, for the first time, the significant inhibitory effect of *N. miranda* extract on elastase, suggesting its potential to prevent elastin degradation, loss of skin elasticity, and the formation of wrinkles.

### 2.2. Anti-Tyrosinase Activity of N. miranda Extract

**Figure 2:**
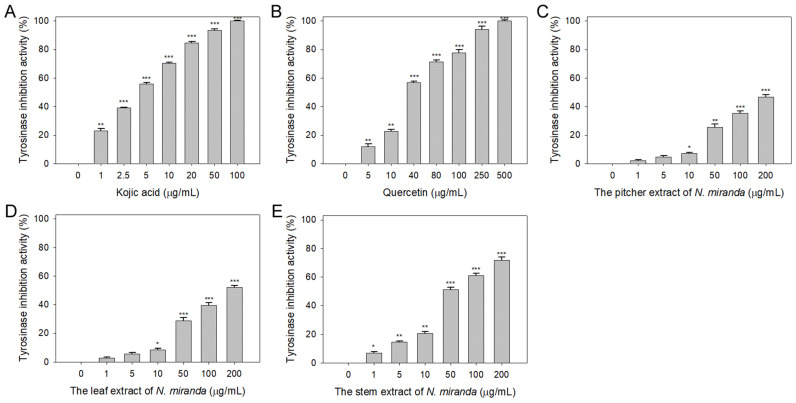
Inhibition of tyrosinase activity by *N. miranda* extract. The inhibitory effects were investigated on tyrosinase activity by (**A**) KA, (**B**) Que, and *N. miranda* extracts from the (**C**) pitcher, (**D**) leaf, and (**E**) stem. L-DOPA was employed as the substrate. KA and Que were utilized as positive controls, while 10% DMSO was used as a negative control (representing 0 μg/mL of *N. miranda* extract). Levels of statistical significance are indicated by * *p* < 0.05, ** *p* < 0.01, and *** *p* < 0.001 in comparison to the control group.

Beyond elastase, melanin synthesis is a key factor in addressing skin aging, with tyrosinase playing an essential role as the primary enzyme in melanogenesis [40,41]. Lowering tyrosinase activity can diminish melanin production, hence the potential inhibitory effect of *N. miranda* extracts on tyrosinase was explored in this study. The capability of *Nepenthes* plant extracts to curb tyrosinase activity had not been previously established. The tyrosinase-inhibiting properties of the extracts (concentrations 0–100 μg/mL) were assessed utilizing the modified dopachrome method, with 3,4-dihydroxy-L-phenylalanine (L-DOPA) as the substrate. Kojic acid (KA) and quercetin (Que) served as positive controls. At a concentration of 10 μg/mL, KA (Figure 2A), Que (Figure 2B), and *N. miranda* extracts from the pitcher (Figure 2C), leaf (Figure 2D), and stem (Figure 2E) exhibited inhibitory effects at rates of 70%, 23%, 7%, 9%, and 21%, respectively. The IC_50_ values were determined to be 3.94 ± 0.32 for KA, 33.64 ± 2.00 for Que, 180.57 ± 0.75 for the leaf, and 48.33 ± 2.92 μg/mL for the stem extract (Table 1). The IC_50_ for the pitcher extract was not ascertainable due to its minimal inhibitory action. These findings mark the first instance of demonstrating that *N. miranda* extracts, particularly the stem extract, can counteract tyrosinase activity, potentially offering protection against the formation of dark pigments and providing therapeutic avenues for treating skin hyperpigmentation and lightening the skin.

### 2.3. Anti-Hyaluronidase Activity of N. miranda Extract

**Figure 3:**
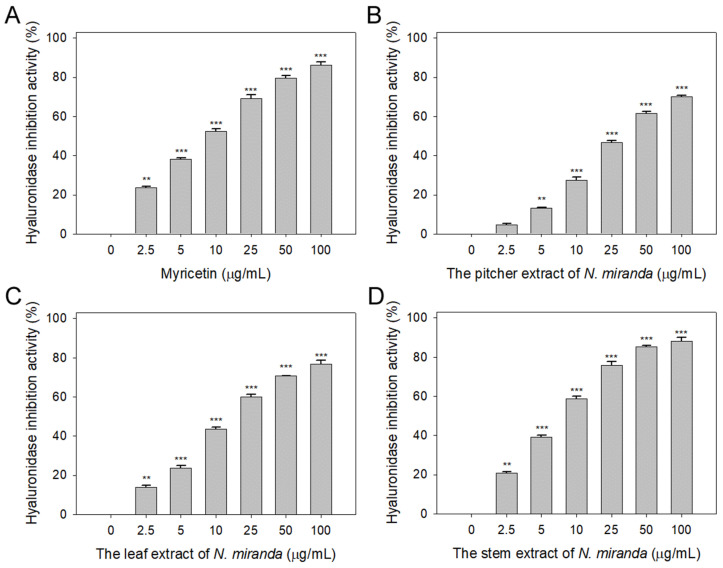
Inhibition of hyaluronidase activity by *N. miranda* extract. The inhibitory effects were investigated on hyaluronidase activity by (**A**) Myr, and *N. miranda* extracts from the (**B**) pitcher, (**C**) leaf, and (**D**) stem. Hyaluronic acid was employed as the substrate. Myr was utilized as a positive control, while 10% DMSO was used as a negative control (representing 0 μg/mL of *N. miranda* extract). Levels of statistical significance are indicated by ** *p* < 0.01 and *** *p* < 0.001 in comparison to the control group.

Prior to this study, the potential of *Nepenthes* plant extracts to inhibit hyaluronidase activity remained unexplored. Hyaluronic acid plays a pivotal role in maintaining moisture, providing a supple, hydrated texture to the body, and mitigating dryness, roughness, and wrinkles. Therefore, inhibitors of hyaluronidase may have significant implications in preserving skin moisture and texture [42]. Accordingly, we evaluated the hyaluronidase-inhibiting properties of extracts from *N. miranda*’s pitcher, leaf, and stem (concentrations ranging from 0–100 μg/mL), extracted using 100% ethanol. Hyaluronic acid was used as the substrate, with myricetin (Myr) as a positive control (0–100 μg/mL). At a concentration of 10 μg/mL, Myr (Figure 3A), and the extracts from the pitcher (Figure 3B), leaf (Figure 3C), and stem (Figure 3D) of *N. miranda* exhibited inhibitory effects at rates of 52%, 28%, 44%, and 59%, respectively. The IC_50_ values were calculated to be 9.52 ± 0.27 μg/mL for Myr, 31.67 ± 2.96 μg/mL for the pitcher, 16.18 ± 1.03 μg/mL for the leaf, and 7.89 ± 0.64 μg/mL for the stem extract (Table 1). These results, for the first time, highlight the considerable inhibitory potential of *N. miranda* extract against hyaluronidase, underscoring its potential role in maintaining hydration and preventing the signs of aging in the skin.

### 2.4. Cytotoxic Activity of the Ethanol Extract of Nepenthes miranda on H838 Lung Adenocarcinoma Cells

**Figure 4:**
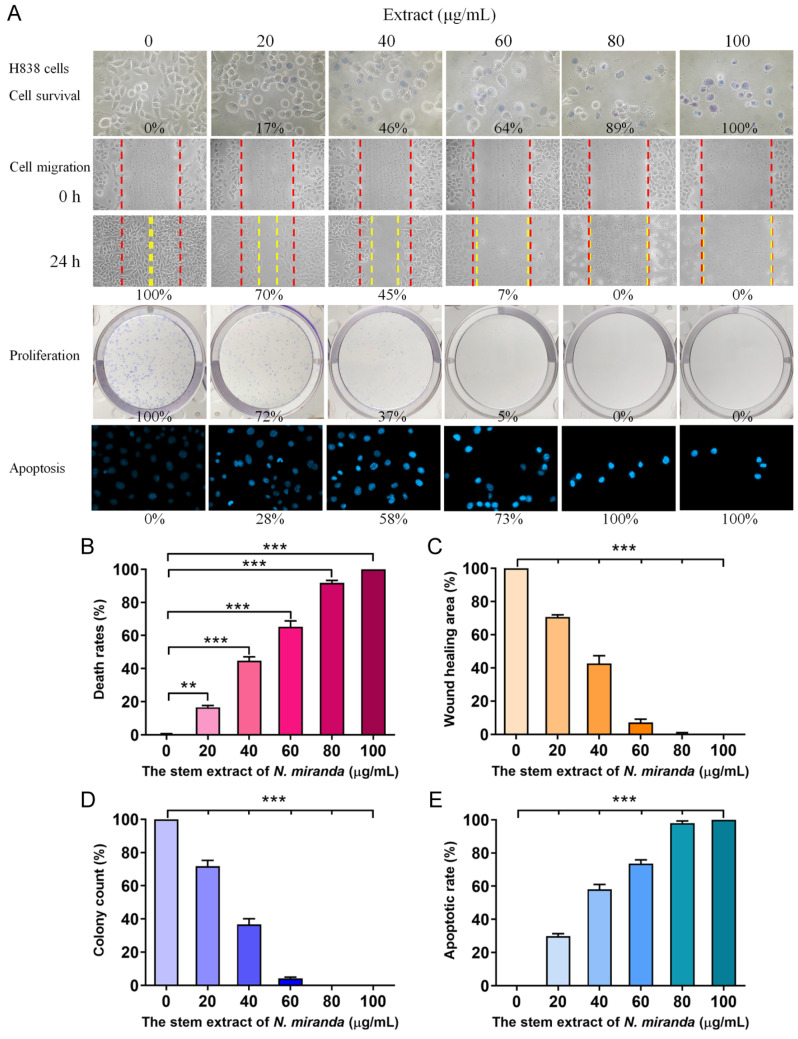
Anticancer potential of *N. miranda* stem extract on H838 cells. (**A**) The effect of *N. miranda* stem extract on H838 cell survival, migration, proliferation, and nuclear condensation. (**B**) Trypan blue exclusion assay showing H838 cell viability following treatment with various concentrations of *N. miranda* extract. (**C**) Wound healing assay depicting the migration of H838 cells treated with different concentrations of *N. miranda* extract. Images were captured immediately and 24 h post-treatment. (**D**) Clonogenic assay assessing the proliferative and colony-forming potential of H838 cells pre-treated with varying concentrations of *N. miranda* extract. (**E**) Hoechst staining illustrating apoptosis and DNA fragmentation in H838 cells at varying concentrations of *N. miranda* extract. Statistical significance is denoted by ** *p* < 0.01 and *** *p* < 0.001 when compared to the control group.

To delve deeper into the cytotoxic effects on cancer cells, particularly H838 human lung carcinoma cells, we utilized the stem extract of *N. miranda* prepared with 100% ethanol to explore its anticancer capabilities. These capabilities encompass cell viability, cell migration inhibition, suppression of cell proliferation, and DNA fragmentation indicative of apoptosis (Figure 4). The anti-H838 efficacy of *N. miranda* extracts was found to follow the order: stem > leaf > pitcher. Consequently, our research primarily focused on investigating the properties of the stem extract. The H838 cell line, derived from the lung tissue of a 59-year-old White male diagnosed with stage 3B adenocarcinoma and displaying epithelial morphology, served as the model for these investigations [43]. For a comprehensive overview, the collective results of these analyses are aggregated in Figure 4A. Initially, we focused on the impact of *N. miranda* extract on H838 cell viability (Figure 4B), utilizing the trypan blue staining assay to determine cytotoxicity. This method is predicated on the concept that viable cells will exclude trypan blue due to their intact membranes, whereas non-viable cells will absorb the dye, thus facilitating the distinction and counting of viable (clear) and non-viable (blue-stained) cells. H838 cell cultures in 96-well plates were subjected to varying concentrations of the extract. The extract stock (20 mg/mL) was diluted with culture medium to obtain the desired experimental concentrations, and cells were incubated with these dilutions or with culture medium containing 0.2% DMSO as the control. Our results demonstrated a pronounced cytotoxic effect of the *N. miranda* extract on H838 cells, with a dose-dependent increase in mortality rates at increasing extract concentrations. Specifically, exposure to 0, 20, 40, 60, 80, and 100 μg/mL of *N. miranda* extract led to 0%, 17%, 46%, 64%, 89%, and 100% cell mortality, respectively (Figure 4B). The sharp differentiation in cell survival between treated and control groups highlights the significant cytotoxic potential of *N. miranda* extract on this cancer cell line. The observed dose-dependent cytotoxicity suggests the presence of active components in the extract that are highly effective in promoting cell death.

### 2.5. The Extract of N. miranda Inhibited the Migration of H838 Cells

The *N. miranda* extract also displayed notable suppressive effects on the migration of H838 cells, which is a critical aspect of cancer metastasis. These effects were monitored using a wound healing assay, a reliable in vitro method to evaluate the migratory response of cells in a two-dimensional framework. For this assay, a wound was introduced into a dense layer of H838 cells, after which various concentrations of *N. miranda* extract were applied. The resultant gap acts as a stimulus for the adjacent cells to move and fill the void. Sequential images taken immediately and 24 h after the treatment of the extract showed a marked reduction in the migration rate as the extract’s concentration was stepped up. Specifically, incubation with 0, 20, 40, 60, 80, and 100 μg/mL of *N. miranda* extract resulted in a 0%, 30%, 55%, 93%, 100%, and 100% reduction in wound closure, respectively (Figure 4C). The decline in cell movement with increasing extract dosage was statistically significant, indicating a strong anti-metastatic effect. The halt in cell migration at concentrations of 80 μg/mL and above demonstrates the extract’s potential in curtailing cancer cell dissemination, a crucial aspect of cancer progression. Therefore, *N. miranda* extract may have valuable applications in curbing cancer metastasis, suggesting its therapeutic potential in cancer treatment strategies.

### 2.6. The Extract of N. miranda Inhibited the Proliferation of H838 Cells

The suppressive action of *N. miranda* extract on the proliferation of H838 cells was evaluated with the clonogenic assay. This assay is a cornerstone for assessing the ability of individual cells to grow and form colonies, thus serving as an indicator of cell survival and proliferation capabilities. The data from the clonogenic assay reveal a substantial reduction in both the quantity and size of colonies with increasing extract doses, indicating a significant impact on cell survival and proliferative potential. Specifically, the application of the extract at 0, 20, 40, 60, 80, and 100 μg/mL concentrations led to a graded inhibition of growth and colony formation in H838 cells by 0%, 28%, 63%, 95%, 100%, and 100%, respectively (Figure 4D). A concentration of 80 μg/mL achieved a complete cessation of cell proliferation, suggesting the extract’s efficacy in blocking the clonal expansion of H838 cells. The marked reduction in colony growth highlights its potential role of the *N. miranda* extract in impeding cancer cell proliferation. Such antiproliferative effects are vital for any agent intended to diminish cancer cell viability and prevent their dissemination. Consequently, the *N. miranda* extract may contain active compounds that may disrupt critical pathways for cell growth and replication, offering a viable direction for future investigations into its utility as a cancer therapeutic.

### 2.7. The Extract of N. miranda Induced Apoptosis of H838 Cells

Our findings indicate that *N. miranda* extract induces apoptosis in H838 cells, as visualized with Hoechst staining. Hoechst 33342, the stain employed in our assay, is a cell-permeant dye that stains the nuclei of all cells. It has a pronounced affinity for the condensed chromatin of apoptotic cells, resulting in a more intense fluorescence compared to non-apoptotic cells. Quantitative analysis of DNA fragmentation, a hallmark of apoptosis, showed that treatment with 0, 20, 40, 60, 80, and 100 μg/mL concentrations of the extract corresponded to 0%, 28%, 58%, 73%, 100%, and 100% increases in DNA fragmentation, respectively (Figure 4E). Morphological signs of apoptosis, such as chromatin condensation and nuclear fragmentation, became increasingly evident with higher concentrations of *N. miranda* extract, indicating a dose-responsive escalation in apoptotic cell death. These apoptotic features are detectable with the intensified blue fluorescence of Hoechst dye binding to the apoptotic chromatin. The induction of complete apoptosis at concentrations of 80 μg/mL and above highlights the strong pro-apoptotic capacity of the extract. Thus, the *N. miranda* extract appears to possess bioactive constituents that may trigger specific apoptotic pathways, offering insights into the extract’s mechanism of action and its potential for cancer therapy.

### 2.8. The Extract of N. miranda Caused DNA Damage in H838 Cells

**Figure 5:**
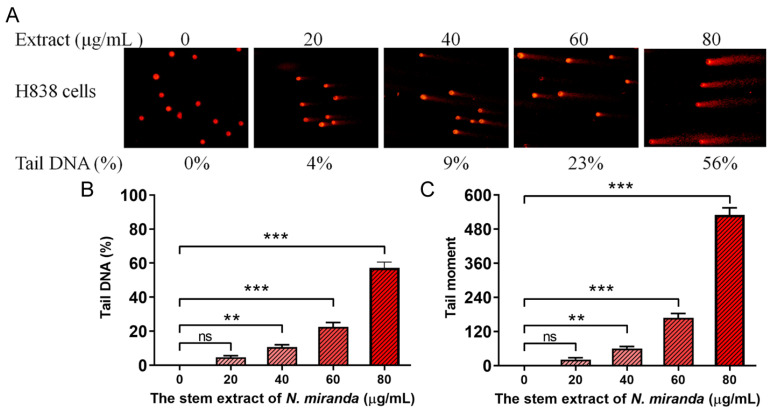
DNA damage induced in H838 cells by *N. miranda* extract. (**A**) Comet assay results display a significant escalation in DNA damage as the concentration of *N. miranda* extract increases. (**B**) A marked increase in comet tail density and (**C**) an extension in comet tail length were observed, reflective of a concentration-dependent rise in DNA damage. Levels of statistical significance are indicated by ** *p* < 0.01 and *** *p* < 0.001 in comparison to the control group. ns—non-significant.

Our research revealed that *N. miranda* extract induces DNA damage in H838 cells, a finding that was demonstrated using the comet assay. This assay, known for its sensitivity in detecting DNA strand breaks at the single-cell level (single-cell gel electrophoresis) [44], showed a distinct increase in DNA damage with higher concentrations of the extract. In the process, H838 cells encapsulated in agarose on a slide underwent lysis, and subsequent electrophoresis produced the characteristic ‘comet’ tails indicative of DNA fragmentation, which were more pronounced in cells treated with *N. miranda* extract compared to controls (Figure 5A). The assay images display a spectrum of DNA damage among the cells, from minimal, evidenced by short or absent comet tails, to severe, indicated by longer tails. These comet tails provide a visual and measurable indicator of the extent of DNA fragmentation. Specifically, treatment with 0, 20, 40, 60, and 80 μg/mL of the extract resulted in 0%, 4%, 9%, 23%, and 56% tail DNA, respectively (Figure 5B). The tail moments in H838 cells, induced by the extract, also exhibited a dose-dependent increase (Figure 5C). The extract’s treatment led to a noticeable increment in both the comet tails’ length and density, indicating a concentration-dependent increase in DNA damage. This kind of damage often leads to cell death, particularly via apoptotic mechanisms, and serves as an essential marker of the extract’s genotoxic capabilities. The induction of DNA strand breaks at elevated extract doses reveals potent cytotoxic and genotoxic activities, pointing to a mode of action that compromises the genetic material within the cancer cells. The ability of the extract to cause such genotoxicity is central to its anticancer effects and offers significant insights into its potential use in cancer therapy by promoting cell death via DNA damage.

### 2.9. Gas Chromatography–Mass Spectrometry (GC–MS) Analysis

In light of the anti-H838 activities and anti-skin-aging potential of *N. miranda* extract, we conducted a gas chromatography–mass spectrometry (GC–MS) analysis to identify the predominant compounds within the extract. The spectral data generated were compared with the NIST 2011 and Wiley 10th edition mass spectral libraries, allowing for the tentative identification of the compounds. Compounds with a similarity index (SI) greater than 800 were considered for identification. Accordingly, the top 15 compounds, each constituting more than 0.5% of the extract, were identified as follows: stigmast-5-en-3-ol (11.7%), plumbagin (10.9%), hexadecanoic acid (3.4%), hexadecanoic acid, 2-hydroxy-1-(hydroxymethyl)ethyl ester (2.4%), catechol (2.3%), oleic acid (2.0%), pyrogallol (2.0%), 13-docosenamide (1.7%), stigmasterol (1.7%), 3-methoxycatechol (1.5%), 1-eicosanol (1.2%), glyceryl monostearate (1.2%), linoleic acid (1.1%), stearic acid (0.8%), and heptadecanol (0.5%). It is conceivable that one or more compounds in the stem extract of *N. miranda*, extracted with 100% ethanol, may act as potential anticancer and anti-skin-aging agents, either individually or synergistically.

### 2.10. The Stem Extract of N. miranda Suppressed Carcinoma Cell Proliferation by Inducing G2 Cell-Cycle Arrest

**Figure 6:**
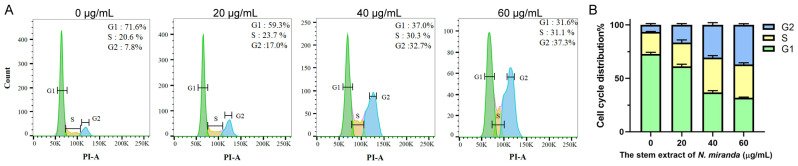
(**A**) Alteration of cell cycle progression by *N. miranda* stem extract in H838 cells. H838 cells underwent treatment with a control solution (0.1% DMSO) or with *N. miranda* stem extract at specified concentrations for 24 h and were subsequently fixed in 70% ethanol overnight. The cells were then stained with propidium iodide (PI) for 30 min before analysis via flow cytometry. (**B**) The cell cycle distribution.

The progression of the cell cycle in H838 cells, upon treatment with *N. miranda* stem extract, was assessed using flow cytometry (Figure 6A). Treatment with the extract at 20, 40, and 60 µg/mL concentrations resulted in an increase in the G2 phase cell population, from 7.8% in untreated cells to 17.0%, 32.7%, and 37.3% in treated cells, respectively, indicating a concentration-dependent accumulation in the G2 phase (Figure 6B). This shift implies a disruption in the normal progression of the cell cycle, which is crucial for cell division. The enrichment in the G2 phase suggests that the *N. miranda* stem extract may exert its anticarcinogenic effects by inducing G2 phase arrest, thereby inhibiting the proliferation of carcinoma cells.

### 2.11. Co-Treatment of N. miranda Stem Extract with the Anticancer Drug 5-Fluorouracil (5-FU) against H838 Cells

**Figure 7:**
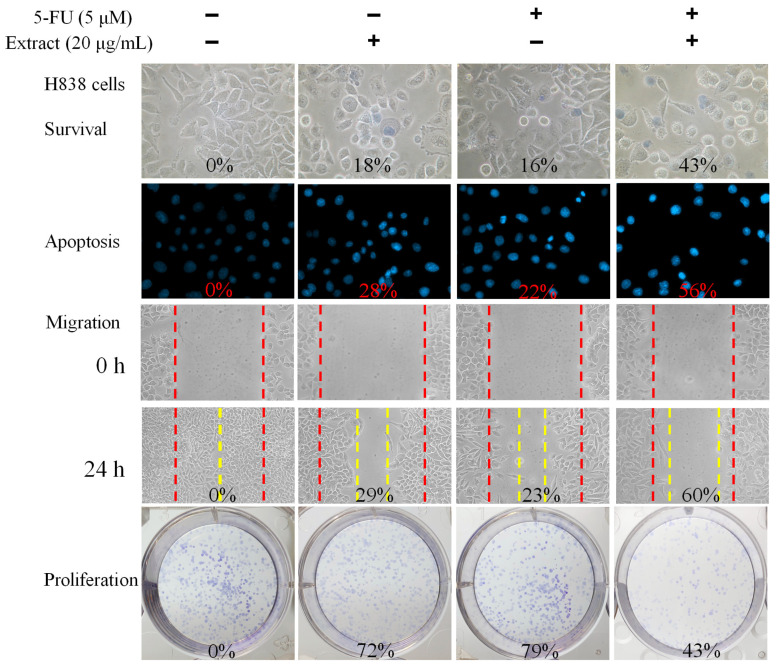
Synergistic anticancer effects of *N. miranda* stem extract and 5-FU on H838 Cells. The combined impact of *N. miranda* stem extract (20 μg/mL) and 5-FU (5 μM) on the survival, migration, proliferation, and apoptosis of H838 cells was assessed. Evaluations were conducted using trypan blue dye exclusion staining for cell viability, Hoechst staining for apoptosis detection, a wound-healing assay for migration analysis, and a clonogenic assay for proliferative capacity. The outcomes of the combination treatment suggest that 5-FU, when used alongside *N. miranda* stem extract, may enhance therapeutic efficacy against cancer.

We also evaluated the combined effect of *N. miranda* stem extract and the chemotherapeutic agent 5-fluorouracil (5-FU) on H838 carcinoma cells (Figure 7). 5-FU, an FDA-approved drug [45], is known for its effectiveness in treating various cancers and has been shown to work synergistically with natural compounds such as myricetin [46], sinomenine [47], lapatinib [48], and plumbagin [22,23] in cancer treatment. Hence, we investigated the potential enhanced efficacy of 5-FU when used in combination with *N. miranda* stem extract against H838 cells. For these experiments, we used a 20 μg/mL concentration of *N. miranda* stem extract, which by itself induces minimal cytotoxicity. Treatment of H838 cells with *N. miranda* stem extract, 5-FU at 5 μM, and their combination resulted in cell death rates of 18%, 16%, and 43%, apoptosis rates of 28%, 22%, and 56%, migration reduction of 29%, 23%, and 60%, and proliferation and colony formation inhibition of 28%, 21%, and 57%, respectively. These findings suggest a synergistic cytotoxic effect, as the combination treatment led to increased cell death, enhanced inhibition of migration and proliferation, and greater induction of DNA fragmentation in H838 cells compared to either agent alone. These results propose that 5-FU could be effectively paired with *N. miranda* stem extract to potentially improve cancer treatment outcomes. However, such synergistic effects need to be validated through further experimental and clinical research.

### 2.12. Inhibition of Human RPA32 by N. miranda Extracts

**Figure 8:**
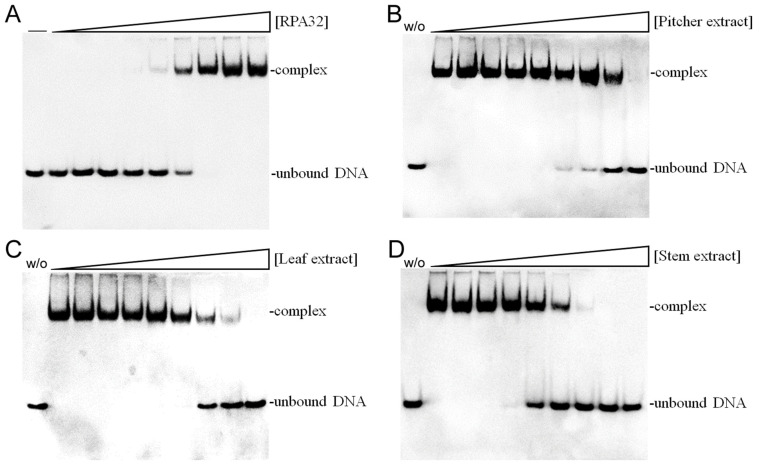
Inhibition of the ssDNA-binding activity of huRPA32 by different extracts of *N. miranda*. (**A**) Binding of huRPA32 to ssDNA dT25. Purified huRPA32 (0, 0.32, 0.63, 1.25, 2.5, 5, 7.5, 10, 20, and 40 μM) was incubated with biotin-labeled ssDNA dT25 and the binding was analyzed with EMSA. The binding constant ([Protein]_50_) of huRPA32 was quantified through linear interpolation based on the protein concentrations. The inhibitory effects were investigated on the DNA-binding activity of huRPA32 by *N. miranda* extracts (0, 0, 7.6, 15.1, 31.3, 62.5, 125, 250, 500, 1000 μg/mL) from the (**B**) pitcher, (**C**) leaf, and (**D**) stem. An amount of 1% DMSO was used as a negative control (representing 0 μg/mL of *N. miranda* extract). The “w/o” denotes the absence of huRPA32 and the extract during the incubation with the DNA dT25.

Given the notable anti-proliferative effects of *N. miranda* stem extract, it is possible that it contains bioactive compounds capable of disrupting key cellular growth and replication pathways. This potential positions it as a promising candidate for further exploration as a cancer treatment. Accordingly, we investigated the possibility of human RPA32 (huRPA32), a critical protein for DNA replication due to its role in binding single-stranded DNA, being a target for inhibition by *N. miranda* stem extract. Considering huRPA32’s necessity in cell division and the fact that normal cells do not perpetually undergo rapid replication, targeting huRPA32 could be an effective strategy for anticancer therapy. Electrophoretic mobility shift analysis (EMSA) was used to evaluate the binding activity of recombinant huRPA32 (concentrations 0–40 μM), which was expressed and purified from *E. coli* (Figure 8A). The principle behind EMSA is that the formation of stable protein–DNA complexes will result in reduced electrophoretic mobility compared to unbound DNA. In this study, the deoxythymidine homopolymer dT25 was used. Incubation with dT25 led to a notable band shift, indicative of stable complex formation. The EMSA titration curve allowed the determination of the midpoint value for ssDNA-binding ([Protein]_50_) of huRPA32 to dT25 as 6.3 ± 0.5 μM. Subsequently, a concentration of 10 μM huRPA32, which effectively binds to dT25, was selected to screen various concentrations (0–1000 μg/mL) of *N. miranda* plant extracts for inhibitory activity. At 250 μg/mL, the extracts from the pitcher (Figure 8B), leaf (Figure 8C), and stem (Figure 8D) of *N. miranda* inhibited huRPA32 activity by 14%, 56%, and 89%, respectively. The IC_50_ values were determined to be 706.6 ± 32.6 μg/mL for the pitcher, 231.4 ± 12.5 μg/mL for the leaf, and 101.6 ± 6.3 μg/mL for the stem extract (Table 1). Therefore, it appears that certain components within the stem extract of *N. miranda*, extracted using 100% ethanol, might act individually or synergistically as potent inhibitors of huRPA32.

### 2.13. Molecular Docking of huRPA32

**Figure 9:**
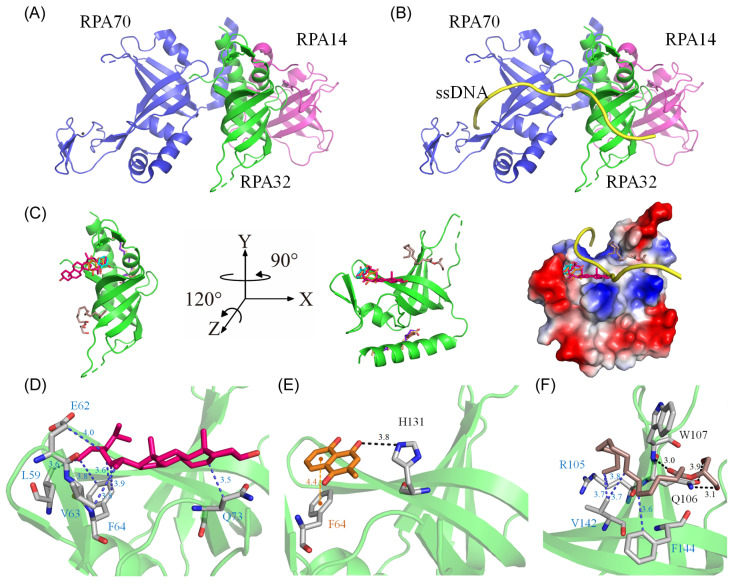
Molecular docking analysis of huRPA32. (**A**) The crystal structure of huRPA32 in complex with huRPA70 and huRPA14 (PDB ID 1L1O), with huRPA70 colored blue, huRPA32 green, and huRPA14 light magenta. (**B**) The modeled huRPA–ssDNA complex, constructed by manually superimposing the apo-huRPA structure with the PaRPA complex (PDB ID 8AAS), assuming similar ssDNA binding mechanisms across species. The ssDNA from the PaRPA complex is colored yellow. (**C**) Docking analysis showing the seven most abundant compounds from the stem extract individually docked into huRPA32: stigmast-5-en-3-ol in hot pink, plumbagin in orange, hexadecanoic acid in purple-blue, hexadecanoic acid, 2-hydroxy-1-(hydroxymethyl)ethyl ester in dark salmon, catechol in cyan, oleic acid in wheat, and pyrogallol in chocolate. Five of these seven compounds targeted various ssDNA binding sites in huRPA32, potentially collectively hindering the binding of ssDNA to huRPA32. The charge distribution pattern is shown to indicate ssDNA binding sites for clarity. (**D**) The binding mode of stigmast-5-en-3-ol, situated within the cavity of the ssDNA-binding surface, engaging in extensive hydrophobic interactions with Leu59, Glu62, Val63, Phe64, and Gln73 of huRPA32. (**E**) The binding mode of plumbagin, which formed a hydrogen bond with His131 and engaged in π-stacking with Phe64. (**F**) The binding mode of hexadecanoic acid, 2-hydroxy-1-(hydroxymethyl)ethyl ester, anchored within the groove for ssDNA binding of huRPA32, formed hydrogen bonds with Gln106 and Trp107, and also interacted hydrophobically with Arg105, Val142, and Phe144.

Our GC-MS analysis identified at least 15 compounds, each constituting over 0.5%, in the stem extract of *N. miranda*, suggesting that certain compounds might contribute to the inhibition of huRPA32. The crystal structure of huRPA32, in complex with huRPA70 and huRPA14 (PDB ID 1L1O) [49], is accessible in the Protein Data Bank (PDB) (Figure 9A), allowing molecular docking studies to predict potential binding sites and calculate binding energies using AutoDock Vina. Although the structure of the huRPA complex with ssDNA remains uncharacterized, the structure of the *Pyrococcus abyssi* RPA (PaRPA)–ssDNA complex has been recently resolved [50]. Consequently, we constructed a model of the huRPA–ssDNA complex (Figure 9B) by manually superimposing the apo-huRPA structure with the PaRPA complex (PDB ID 8AAS), under the assumption of similar ssDNA binding mechanisms across species. For docking analysis, the seven most abundant compounds from the stem extract were individually docked into huRPA32 (Figure 9C). Five of these seven compounds (stigmast-5-en-3-ol, plumbagin, hexadecanoic acid, 2-hydroxy-1-(hydroxymethyl)ethyl ester, catechol, and pyrogallol) targeted various ssDNA binding sites. However, oleic acid and hexadecanoic acid did not. These compounds, by occupying binding sites, may collectively hinder the binding of ssDNA to huRPA32. The binding efficiency of these compounds was ranked as follows (Table 2): stigmast-5-en-3-ol > plumbagin > hexadecanoic acid, 2-hydroxy-1-(hydroxymethyl)ethyl ester > oleic acid > hexadecanoic acid > pyrogallol > catechol. Notably, stigmast-5-en-3-ol and plumbagin, which are the two most prevalent compounds in *N. miranda*’s stem extract, demonstrated strong binding interactions as estimated with AutoDock Vina (Table 2). Stigmast-5-en-3-ol was situated within the cavity of the ssDNA-binding surface and engaged in extensive hydrophobic interactions with Leu59, Glu62, Val63, Phe64, and Gln73 of huRPA32 (Figure 9D). Plumbagin formed a hydrogen bond with His131 and engaged in π-stacking with Phe64 (Figure 9E). Hexadecanoic acid, 2-hydroxy-1-(hydroxymethyl)ethyl ester, anchored within the groove for ssDNA binding of huRPA32, formed hydrogen bonds with Gln106 and Trp107, and also interacted hydrophobically with Arg105, Val142, and Phe144 (Figure 9F). Given the diverse interactions at different ssDNA-binding sites, the inhibitory potential of *N. miranda*’s stem extract against huRPA32 might be attributed to the combined effects of some of these compounds. Nonetheless, this hypothesis requires validation through biochemical and structural studies.

## 3. Discussion

Natural products have attracted significant interest as viable alternatives for treating various diseases due to their multifaceted modes of action on problematic cells [51,52,53,54]. Before this study, the potential of *Nepenthes* plant extracts for anti-skin-aging had not been investigated. *Nepenthes*, recognized for their ethnobotanical uses such as alleviating stomachaches and fevers, show minimal side effects and have demonstrated notable in vitro anticancer and antibacterial properties [55]. This underscores the potential for new therapeutic discoveries and the importance of understanding how *Nepenthes* extracts, such as that from *N. miranda* [56]—a distinctive hybrid of *N. maxima* and *N. northiana* with unique physiological characteristics—act and inhibit specific targets for medical advancement. The goal of this study was to identify natural sources for developing treatments against skin aging (Figure 1, Figure 2 and Figure 3) and cancer (Figure 4, Figure 5, Figure 6 and Figure 7). This research represents the first exploration into the anti-skin-aging potential of *Nepenthes* plants by targeting crucial enzymes involved in skin degradation. The strong inhibitory effects on skin-aging enzymes identified in this study (Table 1), coupled with high polyphenol and flavonoid contents and significant antioxidant activities [23], suggest that *N. miranda* extracts could potentially serve as a natural alternative or complementary therapy. This includes applications such as adjuvants or cosmeceutical ointments for treating symptoms of skin aging. Furthermore, this study identifies RPA32 [36], an essential protein for DNA replication and cell development, as a novel target of *Nepenthes* extract, offering insight into a potential mechanism for inhibiting cancer cell growth by targeting RPA32. The identification of additional plant-derived compounds for anti-aging and anticancer therapies is crucial for further pharmaceutical development.

Skin aging is an inevitable process that affects everyone, yet lifestyle and environmental factors can expedite its onset, leading to prematurely aged skin [57,58]. This research illuminates the comprehensive capability of *N. miranda* extract in mitigating the effects of skin aging. Notably, the extract displays robust inhibitory activity against elastase (Figure 1), tyrosinase (Figure 2), and hyaluronidase (Figure 3), highlighting its effectiveness in preserving skin’s elasticity, minimizing hyperpigmentation, and maintaining moisture and softness. Particularly impressive are its anti-elastase and anti-hyaluronidase properties (Table 1), where the IC_50_ values for *N. miranda*’s stem extract surpassed those of known positive controls EGCG and Myr. Our GC–MS analysis identified at least 15 compounds, each constituting over 0.5%, in the stem extract of *N. miranda*, suggesting that certain compounds might contribute to the strong inhibition of elastase and hyaluronidase. These results underscore *N. miranda* extract’s potential in anti-aging skin care products and open avenues for further investigation into its bioactive constituents as innovative enzyme inhibitors relevant to the skin aging process.

Elastase, a protease enzyme, plays a critical role in breaking down elastin, which is crucial for skin elasticity due to its ability to snap back into shape. Inhibiting elastase can thus prevent the loss of skin elasticity and the formation of wrinkles. Our findings indicate that *N. miranda*’s stem extract is a strong inhibitor of elastase. Although the significant presence of compounds like stigmast-5-en-3-ol (11.7%) and plumbagin (10.9%) in the extract has yet to be explored for their inhibitory effects, hexadecanoic acid (also known as palmitic acid; 3.4%), identified in the extract, is already recognized as a moderate elastase inhibitor [59,60]. The marked anti-elastase activity observed in *N. miranda*’s stem extract could be attributed to a synergistic interaction among the compounds present rather than solely to the action of hexadecanoic acid. Nonetheless, this hypothesis regarding the collective activity of the identified compounds within the stem extract requires further experimental validation.

Hyaluronic acid is crucial for maintaining the body’s hydration, providing smoothness, and ensuring lubrication by binding water molecules. Inhibiting hyaluronidase, therefore, helps to prevent the loss of this moisture. Key components of *N. miranda*’s stem extract, particularly plumbagin [61] and stigmasterol [62], have been identified as potential inhibitors of hyaluronidase. Consequently, the notable anti-hyaluronidase effect of the stem extract may stem from the action of these compounds, possibly in conjunction with other constituents. It merits further investigation to ascertain if *N. miranda*’s stem extract contains any new substances that inhibit hyaluronidase, which could be beneficial for cosmeceutical uses. Our laboratory is currently exploring the hyaluronidase inhibitory effects of these and other active ingredients found in the stem extract of *N. miranda*.

The significant cytotoxic impact of *N. miranda* stem extract on H838 lung carcinoma cells underscores its promise as an anticancer treatment. The dose-dependent increase in cell mortality, suppression of cell migration and proliferation, and induction of apoptosis (Figure 4) and DNA damage (Figure 5) in H838 cells indicates the presence of bioactive compounds in *N. miranda* stem extract capable of targeting multiple pathways involved in cancer cell growth and survival. Our GC–MS analysis revealed that the primary components of the *N. miranda* stem extract, such as stigmast-5-en-3-ol [63], plumbagin [64], and hexadecanoic acid [65], are known for their anticancer properties, contributing to the extract’s potent cytotoxic effects. Exploring whether the various active components in *N. miranda* stem extract can offer significant polypharmacological and synergistic effects, and determining the optimal ratios for cancer treatment, requires further elucidation. Furthermore, flow cytometry findings suggest that the extract may halt the proliferation of NSCLC H838 cells by causing G2 phase cell-cycle arrest (Figure 6). The G2 cell-cycle checkpoint serves as an essential guardian of the genome in tumor cells and, therefore, targeting the cell cycle proteins and abrogating this checkpoint has emerged as a promising therapeutic strategy against cancer [66]. Accordingly, our laboratory is delving into the specific cellular pathways that lead to G2 arrest in H838 cells, aiming to advance the development of new NSCLC therapies.

Leveraging the anti-proliferative effects of *N. miranda* extract, this study suggests that the extract could influence key pathways involved in cellular growth and replication. Additionally, given its capacity to inhibit the ssDNA-binding activity of bacterial SSB [26], we explored and confirmed the extract’s ability to inhibit the crucial DNA replication protein huRPA32 (Figure 8). This indicates that huRPA32 could be a significant target for the anticancer activity of *N. miranda* extract. Given the structural and functional similarities between huRPA32 and SSB, inhibitors of SSB might also effectively target huRPA32. Our recent discoveries include the identification of natural products such as myricetin, taxifolin, dihydrokaempferol, and rutin as SSB inhibitors [30,31,67]. It remains to be seen whether these flavonoids, along with active compounds found in the stem extract of *N. miranda*, can also inhibit huRPA32, potentially contributing to the development of new chemotherapy agents. Docking studies (Figure 9) have indicated that five compounds in the *N. miranda* extract (stigmast-5-en-3-ol, plumbagin, hexadecanoic acid, 2-hydroxy-1-(hydroxymethyl)ethyl ester, catechol, and pyrogallol) target various ssDNA binding sites, suggesting a collective mechanism that may impede ssDNA’s binding to huRPA32. Therefore, the effectiveness of *N. miranda*’s stem extract against huRPA32 could result from the synergistic action of these identified compounds. Nonetheless, this speculation regarding the collective activity of the identified compounds within the stem extract requires further experimental validation.

Despite the advent of new drug development, 5-FU continues to be a fundamental component in cancer treatment, particularly for cancers of the gastrointestinal tract, breast, head, and neck [45]. Our previous research determined the complexed crystal structures of SSB [68], dihydropyrimidinase (DHPase) [69], and dihydroorotase (DHOase) [70] with 5-FU, suggesting that 5-FU’s mechanism of action extends beyond its primary target, thymidylate synthase, to potentially regulate the activity of these proteins. In this study, we discovered that combining the stem extract of *N. miranda* with 5-FU significantly amplifies its cytotoxic impact (Figure 7). 5-FU disrupts RNA function and DNA synthesis [71], while plumbagin—a prominent compound in *N. miranda*’s stem extract—promotes anticancer effects by elevating intracellular reactive oxygen species (ROS) levels and triggering apoptosis, with ROS activation leading to cell death through a p53-dependent pathway [64]. Moreover, plumbagin’s inhibition of DHPase [23] and DHOase [22] affects cellular DNA base metabolism [72]. Our structural analysis showed plumbagin and 5-FU binding at distinct sites within DHPase’s active site [23,72], indicating that plumbagin’s role in enhancing ROS may significantly boost 5-FU’s chemotherapeutic efficacy by concurrently affecting DHPase and DHOase functions. This synergy between *N. miranda*’s stem extract and 5-FU in cytotoxicity suggests a potential pathway for enhancing treatment effectiveness. Further exploration of this synergistic relationship could reveal optimal treatment combinations and dosages, leading to more precise and potent cancer therapies.

While our study provides novel insights into the anti-aging and anticancer potential of *N. miranda* stem extract, we recognize several limitations. The study is predominantly based on in vitro assays, which, although valuable for initial screening, cannot fully replicate the complexity of living organisms. Future studies involving in vivo models are crucial to validate our findings and to assess the pharmacokinetics, pharmacodynamics, and potential toxicity of the extract in a physiological context.

## 4. Materials and Methods

### 4.1. Materials

All solvents and chemicals were of the highest grade and commercially obtained from Sigma-Aldrich (St. Louis, MO, USA). The *Escherichia coli* strain BL21(DE3) pLysS (Novagen, Cambridge, UK) was used for recombinant protein expression. The H838 cell line was kindly provided by Dr. Kuo-Ting Chang (Tao Yuan General Hospital). H838 cells were maintained in RPMI 1640 supplemented with 10% FBS, 100 units/mL penicillin, and 100 μg/mL streptomycin. Cells were incubated at 37 °C in a 95% air and 5% CO_2_ atmosphere.

### 4.2. Expression and Purification of the Recombinant Protein

The synthesized gene encoding huRPA32 (anticipated to express 270 amino acid residues) was cloned using forward and reverse primers (with underlining indicating the restriction site) huRPA32-EcoRI-N (5′-GGGGAATTCATGTGGAACAGTGGATTCGAAAG-3′) and huRPA32-HindIII-C (5′-GGGAAGCTTTTCTGCATCTGTGGATTTAAA-3′). These primers facilitated the insertion of the gene into the pET21e vector, resulting in the construction of the expression plasmid pET21e-huRPA32. This plasmid was designed to express full-length recombinant huRPA32 in *E. coli*. The pET21e vector, a derivative of the pET21b vector (Novagen Inc., Madison, WI, USA), was modified to avoid the fusion of the N-terminal T7 tag with the gene product. The expressed gene product from pET21e-huRPA32 is expected to have two additional artificial residues, EF, introduced by the EcoRI site at the N-terminus, and a C-terminal His tag (KLAAALEHHHHHH), facilitating the purification of the recombinant protein. This resulting plasmid was introduced into *E. coli* BL21 (DE3) cells, which were cultured in LB medium with 250 μg/mL ampicillin until reaching an OD_600_ of 0.9, under vigorous shaking at 37 °C. Recombinant huRPA32 expression was induced with 1 mM isopropyl thiogalactopyranoside (IPTG) for 12 h at 25 °C. The induced cells were harvested, resuspended in sonication buffer (20 mM Tris–HCl, 0.5 M NaCl, pH 8.0), and lysed via sonication. The pellet was then collected, washed with sonication buffer containing 2 M urea, and harvested again. The resulting pellet was solubilized in sonication buffer with 8 M urea for 12 h at 4 °C. The solution was dialyzed twice daily against a refolding buffer (5 mM imidazole, 20 mM Tris–HCl, 0.5 M NaCl, and 1 mM DTT, pH 7.4). The resulting solution was dialyzed again against a purification buffer (5 mM imidazole, 20 mM Tris–HCl, and 0.5 M NaCl, pH 7.9) for 4 h. The recombinant protein was purified from the soluble supernatant using Ni^2+^-affinity chromatography (HisTrap HP; GE Healthcare Bio-Sciences, Piscataway, NJ, USA). Elution was carried out using an elution buffer (20 mM Tris-HCl, 250 mM imidazole, and 0.5 M NaCl, pH 7.9). The purified protein was then dialyzed against a storage buffer (40 mM Tris-HCl, 50 mM NaCl, pH 7.5) and concentrated to 15 mg/mL for future use. Protein purity was verified by electrophoresis on a 12% SDS-PAGE.

### 4.3. Electrophoretic Mobility Shift Analysis (EMSA)

For the analysis of huRPA32 binding, a biotinylated deoxythymidine (dT) homopolymer dT25 was employed as a standard assay substrate. The ssDNA dT25 was biotinylated at its 5′ end. Purified huRPA32 was incubated with this labeled ssDNA (30 fmol/μL) at various concentrations (0, 0.32, 0.63, 1.25, 2.5, 5, 7.5, 10, 20, and 40 μM). The electrophoretic mobility shift analysis (EMSA) was performed using the LightShift Chemiluminescent EMSA Kit. Briefly, huRPA32 and the DNA substrate dT25 were mixed and incubated for 60 min at 37 °C in a buffer containing 40 mM Tris–HCl (pH 7.5) and 50 mM NaCl. After adding the dye mixture, the reaction samples were resolved on an 8% native polyacrylamide gel using EMSA. Electrophoresis was conducted at 100 V for 1 h in TBE running buffer. The protein–DNA complexes were then electroblotted onto a positively charged nylon membrane (GE, Boston, MA, USA). Cross-linking of the transferred DNA to the membrane was achieved using a UV-light instrument equipped with 312 nm bulbs, with an exposure time of 10 min. Detection of the DNA was carried out using the streptavidin–horseradish peroxidase conjugate and chemiluminescent substrate (Pierce Biotechnology, Waltham, MA, USA). The [Protein]_50_, representing the concentration of protein that bound 50% of the input DNA, was estimated from the EMSA results.

### 4.4. huRPA32 Inhibition

An inhibition assay was conducted with huRPA32 at a concentration of 10 μM, incubated with both dT25 and various concentrations of *N. miranda* extract (0, 7.6, 15.1, 31.3, 62.5, 125, 250, 500, 1000 μg/mL). Following EMSA, a titration curve was generated based on the experimental data. The concentration of *N. miranda* extract required to achieve 50% inhibition (IC_50_) of huRPA32 activity was directly determined from this curve using graphical analysis.

### 4.5. Plant Materials and Extract Preparations

The pitcher, leaf, and stem extracts of *N. miranda* were obtained using 100% ethanol [26]. The plant material, procured from Guoguang Flower Market and Taiwan Provincial Flower Marketing Cooperative, was identified by Dr. Zhong-Bao Zhang in December 2020. The collected samples were dried, cut into small pieces, and then pulverized into a powder. For the extraction process, 1 g of the plant powder was placed into a 250 mL conical flask, to which 100 mL of ethanol was added. The mixture was then shaken on an orbital shaker for 5 h. The resulting extract was filtered through a 0.45 μm filter, and the ethanol was subsequently removed using a hot air circulation oven set at 40 °C. The extracts were stored at −80 °C until needed. The extract powder was dissolved in 20% DMSO to prepare a stock solution at a concentration of 20 mg/mL. For anticancer cell assays, the stock solution was diluted with supplemented culture medium to reach the desired assay concentrations. Cancer cells were then incubated with these extract solutions or with the culture medium containing 0.2% DMSO, serving as the treatment and control groups, respectively. For EMSA, the stock solution was diluted with protein storage buffer (40 mM Tris-HCl, 50 mM NaCl, pH 7.5) to the indicated assay concentrations. The protein was then incubated with these extract solutions or with the storage buffer containing 1% DMSO, also serving as treatment and control groups, respectively.

### 4.6. GC-MS Analysis

The composition of the stem extract of *N. miranda*, obtained through ethanol extraction, was tentatively identified using established methodologies [23]. The analysis was performed using a Thermo Scientific TRACE 1300 Gas Chromatograph coupled with a Thermo Scientific ISQ Single Quadrupole Mass Spectrometer System. The chromatographic separation was achieved on a Rxi-5ms column (30 m × 0.25 mm i.d. × 0.25 μm film). Helium, serving as the carrier gas, was maintained at a constant flow rate of 1 mL/min. The oven temperature program started at 40 °C, held for 3 min, followed by a ramp of 10 °C/min to a final temperature of 300 °C, which was then sustained for 1 min. The injection port temperature was set at 300 °C. The eluted compounds were detected by a quadrupole mass detector, with ionization achieved via the electron ionization method. Operational settings included a quadrupole temperature of 150 °C, a source temperature of 300 °C, electron energy of 70 eV, a detector temperature of 300 °C, an emission current multiplier voltage of 1624 V, and an interface temperature of 300 °C. The mass range scanned spanned from 29 to 650 amu. Relative mass fractions of the individual chemical components were calculated employing the peak area normalization method. Compounds were tentatively identified by comparing the generated spectra with the NIST 2011 and Wiley 10th edition mass spectral libraries. Those with a similarity index (SI) exceeding 800 were considered positively identified and are reported in this study.

### 4.7. Elastase Inhibition

The elastase inhibition assay was conducted in accordance with modified methods from previous studies [19]. The assay was carried out in 200 mM Tris buffer (pH 8.0). Porcine pancreatic elastase was prepared as a 3.33 mg/mL stock solution in the same buffer. The substrate N-Succinyl-Ala-Ala-Ala-*p*-nitroanilide (AAAPVN) was dissolved in 200 mM Tris buffer at a concentration of 1.6 mM. Prior to initiating the reaction with the substrate, the test extracts were incubated with the enzyme for 15 min. The final reaction mixture, with a total volume of 250 μL, comprised the buffer, 0.8 mM AAAPVN, 2 μg/mL elastase, and the test extract at concentrations ranging from 0 to 100 μg/mL. Epigallocatechin gallate (EGCG) at concentrations of 0 to 100 μg/mL served as a positive control. Negative controls utilized 10% DMSO. Absorbance was measured at 405 nm immediately after substrate addition and then continuously over a 20 min period using 96-well plates. The percentage of elastase inhibition was calculated using the formula: Elastase inhibition % = [(A_control − A_sample)/A_control] × 100%.

### 4.8. Tyrosinase Inhibition

The inhibitory activity of tyrosinase was assessed using a modified dopachrome method with L-DOPA as the substrate, based on a spectrophotometric approach [73]. The extracts were initially dissolved in 20% DMSO and then diluted to various concentrations using 0.1 M phosphate buffer (pH 6.8). In each well of a 96-well microtitre plate, 5 μL of the extract was combined with 115 μL of 0.1 M phosphate buffer (pH 6.8), 40 μL of mushroom tyrosinase (200 units/mL), and 40 μL of L-DOPA (2.5 mM). The reaction mixture was incubated for 30 min at 37 °C. Alongside each sample, a blank was included containing all the components except L-DOPA to account for background absorbance. The absorbance of the samples was measured at 475 nm. Kojic acid and quercetin were utilized as positive controls for inhibition, while the negative control comprised 10% DMSO in place of the extracts. The percentage of tyrosinase inhibition was calculated using the formula: Tyrosinase inhibition % = [(A_control − A_sample)/A_control] × 100%.

### 4.9. Hyaluronidase Inhibition

The hyaluronidase inhibitory activity was assessed using a method modified from previous studies [74]. Initially, 25 μL of the test extract (concentration range: 0–100 μg/mL) was pre-incubated with 3 μL of hyaluronidase from bovine testes type I-S (H3506, Sigma-Aldrich, USA) at a concentration of 0.4 U/mL in 20 mM phosphate buffer (pH 7), containing 77 mM sodium chloride and 0.01% bovine serum albumin (BSA), for 10 min at 37 °C. Subsequently, 12 μL of phosphate buffer (300 mM, pH 5.35) was added, and the mixture was incubated for an additional 10 min at 37 °C. Then, 10 μL of hyaluronic acid substrate (0.03% in 300 mM phosphate buffer, pH 5.35) was introduced and incubated for 45 min at 37 °C. The reaction, which involves the decomposition of hyaluronic acid, was halted by adding 100 μL of acidic albumin solution (24 mM sodium acetate, 79 mM acetic acid, and 0.1% BSA, pH 3.75). The mixture was then allowed to stand at room temperature for 10 min, after which the absorbance was measured at a wavelength of 600 nm. Myricetin, at concentrations ranging from 0 to 100 μg/mL, served as a positive control, while 10% DMSO was used for the negative controls. The percentage of hyaluronidase inhibition was quantified using the formula: Hyaluronidase inhibition % = [(A_control − A_sample)/A_control] × 100%.

### 4.10. Trypan Blue Cytotoxicity Assay

Cell death was evaluated using the trypan blue cytotoxicity assay [75]. H838 cells (1 × 10^4^) were incubated with *N. miranda* extract in a 100 μL volume for 24 h to assess the cytotoxic activity of the extract via trypan blue dye exclusion.

### 4.11. Chromatin Condensation Assay

Apoptosis in cancer cells was examined using the Hoechst 33342 staining method [76]. H838 cells were seeded in 96-well plates at a density of 5 × 10^3^ cells per well and allowed to adhere for 16 h before incubation with *N. miranda* extract for 24 h. Afterward, cells were washed with PBS and stained with Hoechst dye (1 μg/mL) in the dark for 10 min. Stained cells were imaged using ImageXpress Pico (Molecular Devices, San Jose, CA, USA) with DAPI filter cubes. Image acquisition and analysis were performed using CellReporterXpress Version 2 software.

### 4.12. Clonogenic Formation Assay

The inhibition of H838 cell proliferation was evaluated using a clonogenic formation assay [77]. H838 cells were seeded at a density of 1 × 10^3^ cells per well in 6-well plates and incubated overnight for attachment. Post-treatment with *N. miranda* extract for 5–7 days, cells were washed with PBS, fixed with methanol, and stained with 0.5% crystal violet for 20 min. The number of colonies was counted under a light microscope to assess proliferation inhibition.

### 4.13. Wound-Healing Assay

H838 cell migration inhibition was investigated using a wound healing assay [78], a technique to assess collective cell migration in two dimensions. A cell-free area was created in a confluent cell monolayer, and the presence of this gap induced cell migration to close the wound. H838 cells were grown in serum-reduced medium for 6 h before a linear wound was created using a pipette tip. After washing with serum-reduced medium, cells treated with *N. miranda* extract for 24 h were assessed for migration ability.

### 4.14. Flow Analysis

Flow cytometry was utilized for cell cycle analysis. H838 cells, treated with DMSO or *N. miranda* extract for 24 h, were harvested with trypsin. These cells were then washed, resuspended in PBS containing 1% FBS, and fixed with cold ethanol (70%). After another wash, cells were incubated in PBS for 5 min, then stained with a PI/RNase solution (PBS, RNase, and 50 μg/mL PI) for 30 min at 37 °C in the dark. Cell cycle distribution was analyzed using a BD FACSCanto II (BD Biosciences, San Jose, CA, USA) and visualized with FlowJo v10 software (Tree Star, Inc., Ashland, OR, USA).

### 4.15. Comet Assay

The comet assay was conducted following a modified method from previous research [44]. Cell pellets were resuspended in PBS and combined with 1% low melting point (LMP) agarose at a 1:10 ratio. Seventy-five μL of this mixture was then spread onto a slide pre-treated to enhance LMP agarose adherence. The slides were placed at 4 °C in the dark for 20 min. After the agarose solidified, slides were immersed in a lysis solution (10 mM Tris, 100 mM EDTA, 2.5 M NaCl, 1% Triton X-100, pH 10) for at least 90 min at 4 °C in a dark room. The excess lysis buffer from the slides was tapped off and the slides were washed twice with neutralization buffer (0.4 M Tris–HCl, pH 7.5) for 5 min each. The slides were placed in a horizontal electrophoresis chamber and covered with the electrophoresis buffer (300 mM NaOH, 1 mM EDTA, pH 13). Electrophoresis was conducted at 1.25 V/cm for 25 min. After electrophoresis, slides were washed with deionized water, dehydrated in 95% ethanol, air-dried, stained with PI (2.5 μg/mL; 50 μL per slide), and covered with cover slips in the dark for 30 min. Comet images were captured using a fluorescence microscope (ZEISS Axio Imager A2) and analyzed with Comet Score™ (TriTek Corp., Somerville, NJ, USA), evaluating at least 50 comets per slide for tail intensity percentage and tail moments.

### 4.16. Binding Analysis Using AutoDock Vina

The interaction between compounds and RPA32 was analyzed using AutoDock Vina [79,80,81]. RPA32’s atomic coordinates (PDB ID: 1L1O) were obtained from the RCSB PDB database. Pre-docking preparations, including charge assignments and volume measurements, were performed using AutoDockTools. The compounds’ 2D structures were sourced from PubChem and translated to .sdf format. Both ligands and the protein target were then prepared as PDBQT files for docking with AutoDock Vina through the PyRx Virtual Screening Tool. Docking results were visualized using PyMOL v2.2.0 software.

### 4.17. Statistical Analysis

Experiments were conducted in triplicate, with results presented as mean ± standard deviation (SD). IC_50_ values and statistical significance (*p* < 0.05) were determined using SigmaPlot version 12.0 and GraphPad Prism5 (GraphPad Software Inc., San Diego, CA, USA), respectively, with one-way ANOVA employed to assess differences between means.

## 5. Conclusions

We explored the anti-skin-aging and anti-RPA32 capabilities of various extracts from *N. miranda*, including the stem, leaf, and pitcher, all extracted using ethanol, a solvent deemed safer for human application. We investigated the cytotoxic effect of the stem extract on H838 cancer cells, focusing on cell survival, apoptosis, migration, proliferation, and DNA damage. The major components of this extract were identified through GC–MS analysis to evaluate their potential for multi-targeted therapeutic actions and synergistic benefits. The findings collectively suggest the promising pharmacological value of *N. miranda*, offering a foundation for its potential inclusion in future drug development.

## Figures and Tables

**Table 1:** Inhibition effects of *N. miranda* extract.

	IC_50_ Value (μg/mL)			
Inhibitor	Elastase	Tyrosinase	Hyaluronidase	RPA32
Stem extract	2.29 ± 0.68	48.33 ± 2.92	7.89 ± 0.64	101.6 ± 6.3
Leaf extract	5.31 ± 0.31	180.57 ± 0.75	16.18 ± 1.03	231.4 ± 12.5
Pitcher extract	8.66 ± 1.15	ND	31.67 ± 2.96	706.6 ± 32.6
KA	–	3.94 ± 0.32	–	–
Que	–	33.64 ± 2.00	–	–
EGCG	4.41 ± 0.34	–	–	–
Myr	–	–	9.52 ± 0.27	–

**Table 2:** Molecular docking analysis of huRPA32.

	Affinity (kcal/mol)	Interaction	Residue (Distance, Å)
Stigmast-5-en-3-ol	−7.2	Hydrophobic	L59 (3.59), E62 (3.98), V63 (3.80), F64 (3.59, 3.83, 3.85), Q73 (3.54)
Plumbagin	−6.2	Hydrogen bond	H131 (3.75)
		π-Stacking	F64 (4.40)
Hexadecanoic acid	−5.1	Hydrophobic	T50 (3.57, 3.72, 3.92), V77 (4.00), D96 (3.66), T98 (3.42), F155 (3.50), H158 (3.65), V162 (3.62), I163 (3.77)
Hexadecanoic acid, 2-hydroxy-	−5.3	Hydrogen bond	Q106 (3.10, 3.86), W107 (3.00)
1-(hydroxymethyl)ethyl ester		Hydrophobic	R105 (3.77), V142 (3.69, 3.73), F144 (3.62)
Catechol	−4.7	Hydrogen bond	E62 (3.74), R133 (2.80), S134 (2.81)
		Hydrophobic	L59 (3.62, 3.74), F64 (3.60, 3.73)
Oleic acid	−5.3	Hydrogen bond	V77 (2.87), I159 (3.77)
		Hydrophobic	T50 (3.61), D96 (3.76), T98 (3.49), I159 (3.55, 3.64), I163 (3.61, 3.71), H166 (3.62)
Pyrogallol	−4.8	Hydrogen bond	V77 (2.89), D96 (2.94)
		Hydrophobic	T50 (3.73), I159 (3.53)

## Data Availability

Data are contained within the article.
